# Providing carbon skeletons to sustain amide synthesis in roots underlines the suitability of *Brachypodium distachyon* for the study of ammonium stress in cereals

**DOI:** 10.1093/aobpla/plz029

**Published:** 2019-05-10

**Authors:** Marlon de la Peña, María Begoña González-Moro, Daniel Marino

**Affiliations:** 1 Department of Plant Biology and Ecology, University of the Basque Country (UPV/EHU), Leioa, Spain; 2 Ikerbasque, Basque Foundation for Science, Bilbao, Spain

**Keywords:** Ammonium assimilation, Asn, carbon metabolism, Gln, monocots, nitrate, nitrogen metabolism, root, TCA cycle

## Abstract

Plants mainly acquire N from the soil in the form of nitrate (NO_3_^−^) or ammonium (NH_4_^+^). Ammonium-based nutrition is gaining interest because it helps to avoid the environmental pollution associated with nitrate fertilization. However, in general, plants prefer NO_3_^−^ and indeed, when growing only with NH_4_^+^ they can encounter so-called ammonium stress. Since *Brachypodium distachyon* is a useful model species for the study of monocot physiology and genetics, we chose it to characterize performance under ammonium nutrition. *Brachypodium distachyon* Bd21 plants were grown hydroponically in 1 or 2.5 mM NO_3_^−^ or NH_4_^+^. Nitrogen and carbon metabolism associated with NH_4_^+^ assimilation was evaluated in terms of tissue contents of NO_3_^−^, NH_4_^+^, K, Mg, Ca, amino acids and organic acids together with tricarboxylic acid (TCA) cycle and NH_4_^+^-assimilating enzyme activities and RNA transcript levels. The roots behaved as a physiological barrier preventing NH_4_^+^ translocation to aerial parts, as indicated by a sizeable accumulation of NH_4_^+^, Asn and Gln in the roots. A continuing high NH_4_^+^ assimilation rate was made possible by a tuning of the TCA cycle and its associated anaplerotic pathways to match 2-oxoglutarate and oxaloacetate demand for Gln and Asn synthesis. These results show *B. distachyon* to be a highly suitable tool for the study of the physiological, molecular and genetic basis of ammonium nutrition in cereals.

## Teaser

Nitrogen fertilization may be harmful for the environment. The loss of nitrate provokes water contamination and nitrogen is also emitted in the form of greenhouse gases that contribute to global warming. Ammonium-based fertilizers are useful in mitigating some of these unwanted environmental effects. Unfortunately, plants often grow less with ammonium compared to nitrate supply. With the overall objective of better understanding plants’ response to ammonium nutrition, in this work we propose the grass *Brachypodium distachyon* as a highly suitable tool for the study of the molecular and genetic basis of ammonium nutrition in cereals. This is of great importance in the context of promoting more environmentally friendly nitrogen fertilization.

## Introduction

Over the last decade, *Brachypodium distachyon* has gained attention as model plant for C3 grasses. Phylogenetically, it lies between rice and wheat, with a high degree of sequence similarity with wheat, and high degree of synteny with most grasses (Brutnell *et al.* 2015). Given *B. distachyon* is not domesticated, it shows great intra-species diversity; its pan-genome containing nearly twice the number of genes found in any individual genome (Gordon *et al.* 2017).

Although many aspects of *Brachypodium* development and responses to biotic and abiotic stresses have been studied, little has been published concerning *Brachypodium* nitrogen (N) signalling and metabolism (Ingram *et al.* 2012; Poiré *et al.* 2014; Barhoumi 2017) and, to our knowledge, no report is available on how *B. distachyon* deals with different N sources. This point is crucial since N is the major mineral nutrient demanded by plants and its availability is yield-limiting in many agronomic soils (Xu *et al.* 2012). Plants take up N mainly in form of ammonium (NH_3_/NH_4_^+^) and nitrate (NO_3_^−^). Nitrate is usually the preferred source but is a source of pollution because anions are readily lost through leaching. Besides, nitrous oxide (N_2_O), one of the strongest greenhouse gases, is emitted during bacterial denitrification (Huérfano *et al.* 2015). Ammonium salts, when combined with nitrification inhibitors, are more stable in the soil and have been proved useful in mitigating some of the unwanted effects of nitrate fertilization (Huérfano *et al.* 2015). Moreover, ammonium nutrition can sometimes confer positive effects on plant performance, for example by increasing sorghum and rice tolerance to osmotic stress (Gao *et al.* 2010; Miranda *et al.* 2016). It has also been suggested that ammonium nutrition may improve the response of some species to high concentrations of atmospheric CO_2_ (Bloom *et al.* 2010). In addition, a frequent characteristic associated with ammonium nutrition is an enrichment with N-containing compounds (Marino *et al.* 2016; Coleto *et al.* 2017). However, ammonium nutrition is also known to decrease plant growth. This is the main symptom of ammonium stress, the so-called ‘ammonium syndrome’ (Liu and Von Wirén 2017). The energetic cost associated with maintaining cytosolic NH_3_/NH_4_^+^ homeostasis, mainly by pumping NH_3_/NH_4_^+^ out of the cytosol and by increasing NH_4_^+^ assimilation, is considered to be one of the major causes of biomass reduction (Britto and Kronzucker 2002; Esteban *et al.* 2016). If the concentration of NH_3_/NH_4_^+^ exceeds the capacity for efflux and assimilation, NH_4_^+^ is, in most species, preferentially accumulated in root cells to avoid damaging the photosynthetic apparatus (Esteban *et al.* 2016). Overall, the study of the metabolic adaptation to ammonium stress is crucial to increase plant N use efficiency while reducing N losses associated with nitrate fertilization.

Ammonium is mainly assimilated via the glutamine synthetase/glutamate synthase (GS/GOGAT) cycle. To sustain GS/GOGAT activity, the tricarboxylic acid (TCA) cycle and its associated routes regulate the continuous supply of carbon skeletons. Indeed, proper management of carbon supply has been shown to be essential for ammonium tolerance (Roosta and Schjoerring 2008; Vega-Mas *et al.* 2015). Although controlling NH_3_/NH_4_^+^ entry/efflux and its assimilation is crucial for NH_4_^+^ homeostasis, ammonium stress is also related to other processes such as pH control, ion imbalance and nitrate signalling (Liu and Von Wirén 2017). The study of the co-ordination and regulation of all these mechanisms is essential to understand fully how they determine the extent of tolerance/sensitivity to ammonium nutrition in a given species or genotype. For instance, there is considerable inter- and intraspecific variability in the extent of ammonium stress amongst grass species such as maize (Schortemeyer *et al.* 1997), rice (Chen *et al.* 2013) and wheat (Wang *et al.* 2016a).

In this work, we undertook a comprehensive physiological and metabolic characterization of *B. distachyon* (reference genotype Bd21) grown with exclusive access to NH_4_^+^ or NO_3_^−^ as N source. We focused on leaf and root carbon metabolism and on nitrogen assimilatory pathways.

## Methods

### Growth conditions and experimental design

*Brachypodium distachyon* Bd21 seeds were sterilized in 100 % ethanol for 1 min, rinsed three times with deionized water and incubated in deionized water for 2 h. Then, seeds were placed in trays filled with a perlite:vermiculite (1:1) mixture moistened with deionized water. After 4 days of stratification at 4 °C in the dark, the trays were transferred to a growth chamber (60/70 % of relative humidity, 23 °C day (14 h) with a light intensity of 350 μmol m^−2^ s^−1^ and 18 °C night (10 h)). Eleven days after sowing, seedlings were transferred to 4.5-L hydroponic tanks (10 plants per tank). The nutrient solution contained 1.15 mM K_2_HPO_4_, 0.85 mM MgSO_4_, 0.7 mM CaSO_4_, 2.68 mM KCl, 0.5 mM CaCO_3_, 0.07 mM NaFeEDTA, 16.5 μM Na_2_MoO_4_, 3.7 μM FeCl_3_, 3.5 μM ZnSO_4_, 16.2 μM H_3_BO_3_, 0.47 μM MnSO_4_, 0.12 μM CuSO_4_, 0.21 μM AlCl_3_, 0.126 μM NiCl_2_ and 0.06 μM KI, pH 6.8. The nitrogen source was (NH_4_)_2_SO_4_ for ammonium-fed plants and Ca(NO_3_)_2_ for nitrate-fed ones. Each N source was supplied at 1 or 2.5 mM of total N. To compare both N sources within each concentration, NO_3_^−^-fed plants were supplied with CaSO_4_ to match the SO_4_^2−^ supplied with the NH_4_^+^. The pH of the solution was checked every 2 days and the nutrient solution replaced every 4 days. Four tanks were set up per treatment; thus, a total of 40 plants were grown per condition. Twenty-four days after transfer to hydroponic conditions, plants were harvested. Shoots and roots were separated and individually weighed. For metabolic measurements, plants grown in the same tank were pooled and immediately frozen in liquid nitrogen, homogenized in a Tissue Lyser (Retsch MM 400) and stored at −80 °C until use.

### Element and metabolite determination

Nitrogen and carbon content was determined with an elemental analyser Flash EA1112 (Thermo Fisher Scientific Inc., Waltham, MA, USA). Chlorophyll was extracted in 80 % aqueous acetone and quantified spectrophotometrically (Arnon 1949). Ammonium was extracted from 50 mg of frozen leaf and root powder as described in Sarasketa *et al.* (2014) and quantified following the phenol hypochlorite method. Nitrate was determined as described in Tschoep *et al.* (2009). Inorganic elements were extracted from 10 mg of lyophilized leaf and root with HNO_3_ and microwave-assisted digestion (Mars6, Vertex, Spain). Quantification was performed using an optical emission spectrophotometer with inductively coupled plasma ICP-OES (Horiba Jobin Yvon, Activa). Amino acids were determined by capillary electrophoresis (PA-800, Beckman Coulter Inc., USA) coupled with laser-induced fluorescence detection (argon laser at 488 nm) as previously described (Orcaray *et al.* 2010). Organic acids were extracted from 150 and 200 mg of frozen leaf and root, respectively, in 1.8 mL of methanol:water:chloroform (4:4:10). After centrifugation, the upper layer was recovered, vacuum dried and the pellet resuspended in 1 mL of ultrapure water that was finally filtered through a 0.22-µm PES filter. Separation and quantification were performed by ion chromatography (Dionex ICS-5000, Thermo Scientific).

### Protein content and enzyme activities determination

Protein was extracted from 100 mg of frozen leaf and root powder with 1 mL extraction buffer as described in Sarasketa *et al.* (2016). Protein was quantified using a Bradford base dye-binding assay (Bio-Rad, Hercules, CA, USA) with bovine serum albumin as a standard. Enzyme activities were determined with a 96-well plate spectrophotometer (BioTek Instruments). Glutamine synthetase (GS), NADH glutamine 2-oxoglutarate aminotransferase (GOGAT), NAD(H)- and NADP(H)-dependent glutamate dehydrogenase (GDH and NADP-GDH, respectively) in its aminating sense, NADP-dependent isocitrate dehydrogenase (ICDH), NAD- and NADP-dependent malic enzymes (ME), malate dehydrogenase (MDH) and phosphoenolpyruvate carboxylase (PEPC) were assayed as described in Coleto *et al.* (2017). Aspartate aminotransferase (AAT) was measured as in Gordon and Kessler (1990).

### RNA extraction and gene expression analysis

RNA extraction was from 25 mg of frozen leaf or root powder with the Nucleospin RNA plant kit (Macherey-Nagel) that includes DNAse treatment. One microgram of RNA was retrotranscribed into cDNA (PrimeScript™ RT; Takara Bio Inc.) and gene expression was determined from 2 µL of cDNA diluted 1:10 in a 15 μL reaction volume using SYBR Premix ExTaq™ (Takara Bio Inc.) in a Step One Plus Real Time PCR System (Applied Biosystems). The PCR programme was 95 °C for 5 min followed by 40 cycles of 94 °C for 15 s and 60 °C for 1 min and a melting curve (40–95 °C with one fluorescence read every 0.3 °C). *ACT3* and *SamDC* were used as housekeeping genes to normalize gene expression. Absence of genomic DNA contamination was checked in all RNA samples. In **Supporting Information—Table S1**, the primers used and their efficiency, calculated with serial cDNA dilutions, are described.

### Phylogenetic analysis

Phylogenetic analysis was done using the programmes included in the Phylogeny.fr tool (Dereeper *et al.* 2008) as follows. Coding sequences multiple alignments were conducted with the Muscle algorithm and refined using G blocks. Phylogeny analysis was done using the bootstrapping procedure with the PhyML software. Finally, TreeDyn was used to visualize the tree.

### Statistical analysis

All the results presented are given as means with standard errors. Data were analysed with SPSS 17.0 (Chicago, IL, USA). Normality and homogeneity of variance were analysed by Kolmogorov–Smirnov and Levene’s tests. The significance of the results was assessed using independent samples *t*-test or two-way ANOVA.

## Results

**Figure 1.**
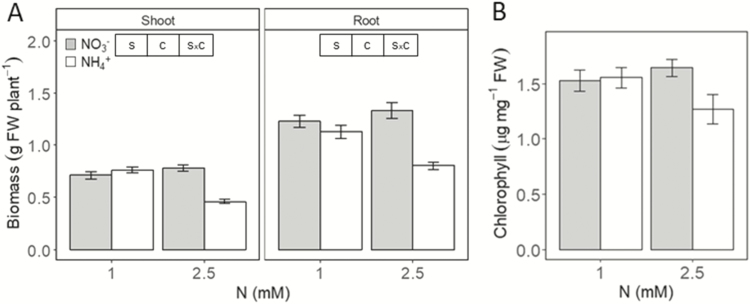
Comparison of *B. distachyon* fresh weight and chlorophyll content in response to N source after 24 days. (A) Plant biomass. (B) Chlorophyll content. Plants were grown with nitrate or ammonium as N source at 1 or 2.5 mM. Columns represent mean ± SE (*n* = 40 for biomass data and *n* = 4 for chlorophyll). Whenever significant differences according to two-way ANOVA, S indicates N source effect; C indicates N concentration effect and S × C indicates interaction effect (*P* < 0.05).

We compared the performance of *B. distachyon* Bd21 growing under the exclusive supply of NH_4_^+^ as N source with that of plants given an exclusive supply of NO_3_^−^ as the control condition. Concentrations of 1 or 2.5 mM of each N source were given. Statistical analyses indicated that biomass was affected by the N source, its concentration and there was also a significant statistical interaction (Fig. 1A). Notably, when plants were grown in 1 mM ammonium growth by roots or shoots was closely similar to that by plants in 1 mM nitrate. When NH_4_^+^ was increased to 2.5 mM, plants showed symptoms of ammonium stress, i.e. a lower biomass compared to plants in 2.5 mM nitrate (Fig. 1A). Chlorophyll content was not affected by the treatment (Fig. 1B). Therefore, 1 mM ammonium represented a non-toxic supply while 2.5 mM was toxic.

**Figure 2.**
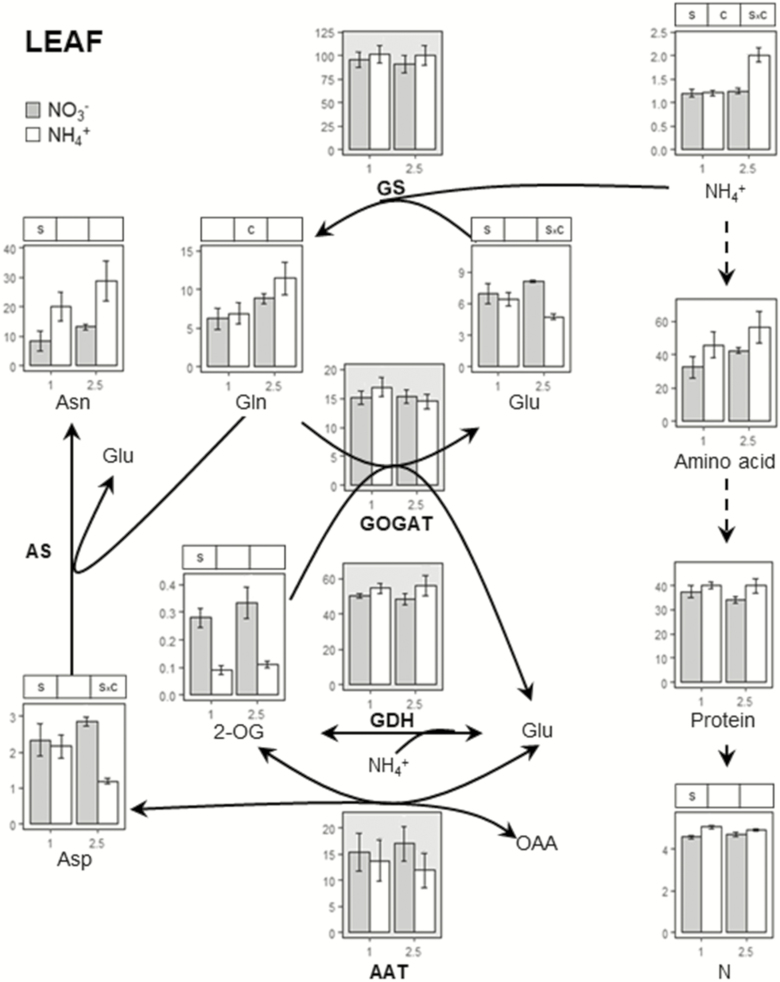
Ammonium assimilation in *B. distachyon* leaf tissue. Plants were grown with nitrate or ammonium as N source for 24 days at 1 or 2.5 mM. Ammonium, total amino acids, Asn, Glu, Gln, Asp and 2-OG content are expressed µmol g^−1^ FW. For enzyme activities, GOGAT, GDH and AAT are expressed as µmol NADH g^−1^ FW h^−1^ and GS as µmol γ-GHM g^−1^ FW h^−1^. Protein content is expressed as mg BSA g^−1^ FW and total N content as % dry wt. Columns represent mean ± SE (*n* = 3–4). Significant differences according to two-way ANOVA are indicated by S for N source effects, C for N concentration effects and S × C for interactions (*P* < 0.05).

**Figure 3.**
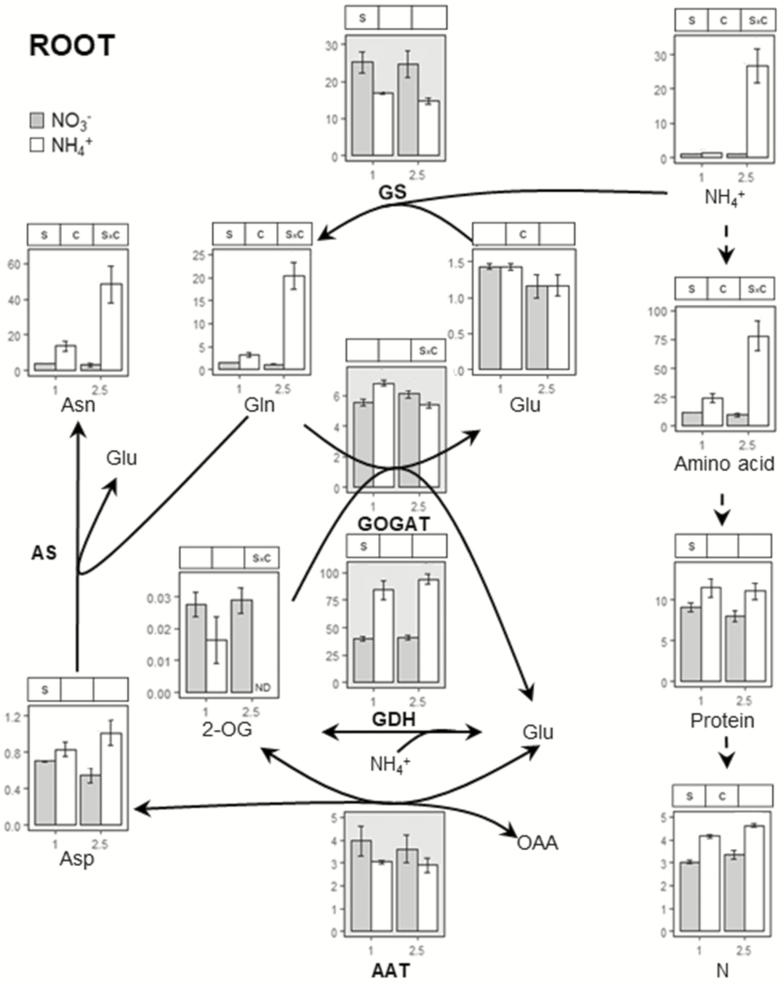
Ammonium assimilation in *B. distachyon* root tissue. Plants were grown with nitrate or ammonium as N source at 1 or 2.5 mM for 24 days. Ammonium, total amino acids, Asn, Glu, Gln, Asp and 2-OG content are expressed µmol g^−1^ FW. For enzyme activities, GOGAT, GDH and AAT are expressed as µmol NADH g^−1^ FW h^−1^ and GS as µmol γ-GHM g^−1^ FW h^−1^. Protein content is expressed as mg BSA g^−1^ FW and total N content as % dry wt. Significant differences according to two-way ANOVA are indicated by S for N source effects, C for N concentration effects and S × C for interactions (*P* < 0.05).

To analyse N metabolism we determined contents of NH_4_^+^, NO_3_^−^, protein, total N, individual amino acids and the activity of enzymes related to N metabolism: GS, GOGAT, AAT and glutamate dehydrogenase (GDH) in both leaf and root (Figs 2 and 3). The accumulation of ammonium (NH_4_^+^) is the most common metabolic marker of ammonium stress. In agreement with biomass and chlorophyll content, NH_4_^+^ content did not accumulate in plants fed with 1 mM ammonium (Figs 2 and 3). However, with 2.5 mM supply, root tissue accumulated *ca.* 25 times more NH_4_^+^ compared to equivalent nitrate nutrition (Fig. 3) whereas in leaf tissue, little NH_4_^+^ accumulated (Fig. 2). As expected, NO_3_^−^ content was affected by the N source with NO_3_^−^ levels being much higher for plants grown in nitrate. Indeed, in plants fed with ammonium NO_3_^−^ content was so low it approached the limit of detection of the methodology used **[see****Supporting Information—Fig. S1****]**.

Asn and Gln were the most abundant amino acids in root and leaf under both NO_3_^−^ and NH_4_^+^, with Glu also being abundant in plants fed with nitrate (Figs 2 and 3; **see****Supporting Information—Tables S2****and****S3**). At 1 mM, differences between NO_3_^−^- and NH_4_^+^-fed plants were small. Exceptions were Asn and Phe contents which were markedly higher in roots of plants grown with ammonium (Fig. 3; **see****Supporting Information—Table S3**). With 2.5 mM NH_4_^+^ supply a large accumulation of NH_4_^+^ in the roots was associated with much increased levels of amino acids **[see****Supporting Information—Table S3****]**, mainly in form of Asn and Gln where contents increased *ca*. 17 and 19 times, respectively (Fig. 3). Overall, total amino acids content was *ca*. 8 times higher compared to nitrate nutrition (Fig. 3).

A substantial N source effect was observed in root GDH (NADH- and NADPH-dependent) and GS enzyme activities (Fig. 3; **see****Supporting Information—Fig. S2A**) under both 1 and 2.5 mM. Glutamate dehydrogenase showed high activity in the root of plants fed with ammonium, while the contrary was the case for GS activity (Fig. 3). The stimulation of N assimilation in plants fed with ammonium was also evident in terms of Gln/Glu and Asn/Asp ratios **[see****Supporting Information—Fig. S3****]**. Importantly, the increase in Gln/Glu and Asn/Asp ratios in the leaves of plants grown with 2.5 mM of ammonium was not only due to Gln and Asn increase but also to a decrease in Glu and Asp (Fig. 2).

**Figure 4.**
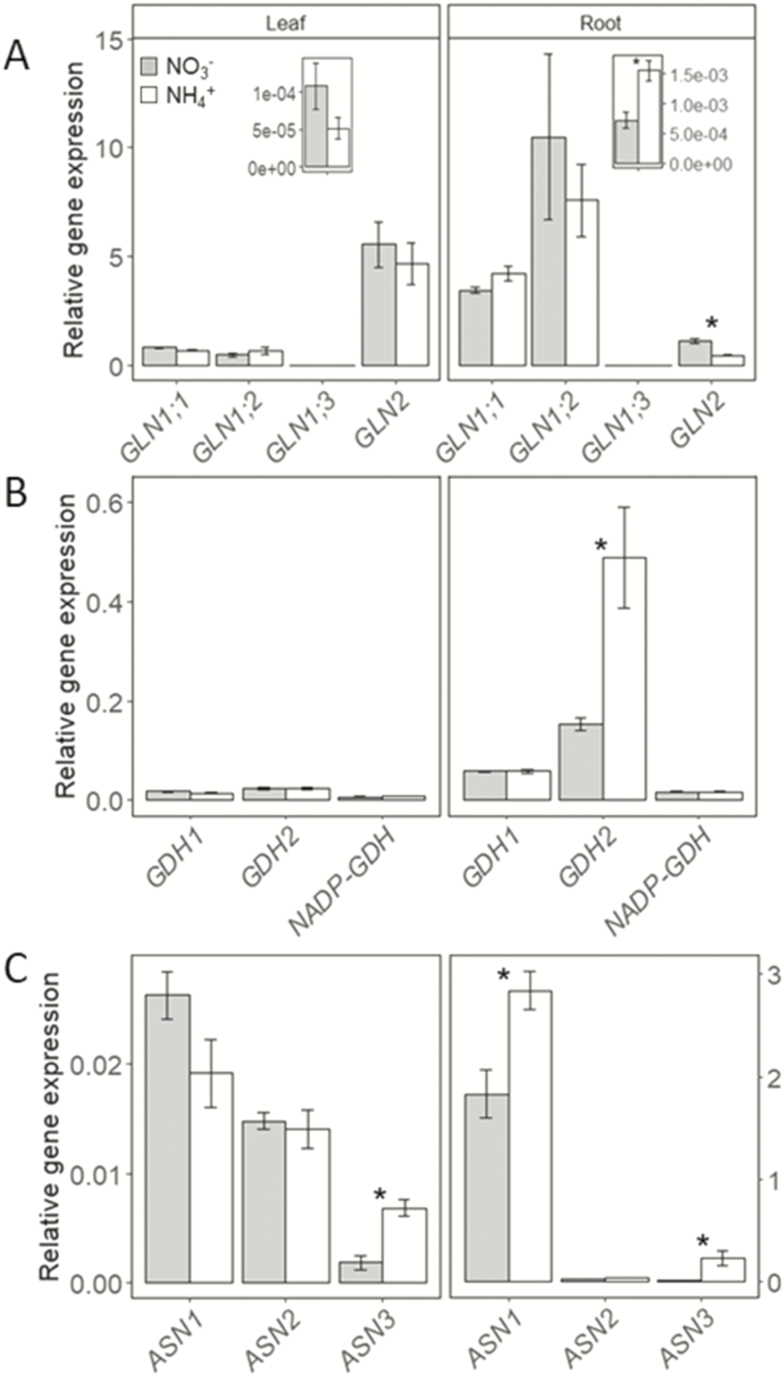
GLN, GDH and ASN gene expression pattern in leaves and roots of plants grown for 24 days with 2.5 mM ammonium or nitrate as exclusive source of nitrogen. (A) *GLN* genes expression. (B) *GDH* genes expression. (C) *ASN* genes expression. The insets in panels A and B show the details for *GLN1;3* expression. Columns represent mean ± SE (*n* = 4). Asterisk (*) indicates significant nitrogen source effect within each nitrogen concentration (*t*-test, *P* < 0.05).

To evaluate the regulation of ammonium assimilation enzymes, we looked for genes encoding GS, GDH and AS in *B. distachyon* Bd21. We carried out BLAST analysis in GenBank (https://www.ncbi.nlm.nih.gov/genbank/) and Phytozome (https://phytozome.jgi.doe.gov/) databases, using available sequences for *Oryza sativa*, *Triticum aestivum* and *Arabidopsis thaliana GS*, *GDH* and *ASN* genes as queries. We found complete sequences for four genes encoding for GS (three GS1 and one GS2), two genes encoding for GDH and one for NADP-GDH and three genes encoding for AS **[see****Supporting Information—Table S1****]**. We performed a phylogenetic analysis for the three families including sequences for *A. thaliana*, *T. aestivum*, *O. sativa*, *Hordeum vulgare* and *Aegilops tauschii***[see****Supporting Information—Figs S4****–****S6****]**. In general, *B. distachyon* genes lie between Triticeae and rice genes. *Arabidopsis* genes were more difficult to position **[see****Supporting Information—Figs S4****–****S6****]**. We analysed gene expression by qPCR in leaf and root tissue of plants grown for 24 days with 2.5 mM nitrate or ammonium supply. In roots, expression of *BdGLN1;3* (Fig. 4A), *BdGDH2* (Fig. 4B), *BdASN1* and *BdASN3* (Fig. 4C) was higher in plants under ammonium nutrition. In contrast, the expression of *BdGLN2* was higher in roots of plants fed with nitrate (Fig. 4A). In leaves, only *BdASN3* expression significantly increased in plants grown with ammonium (Fig. 4C).

**Figure 5.**
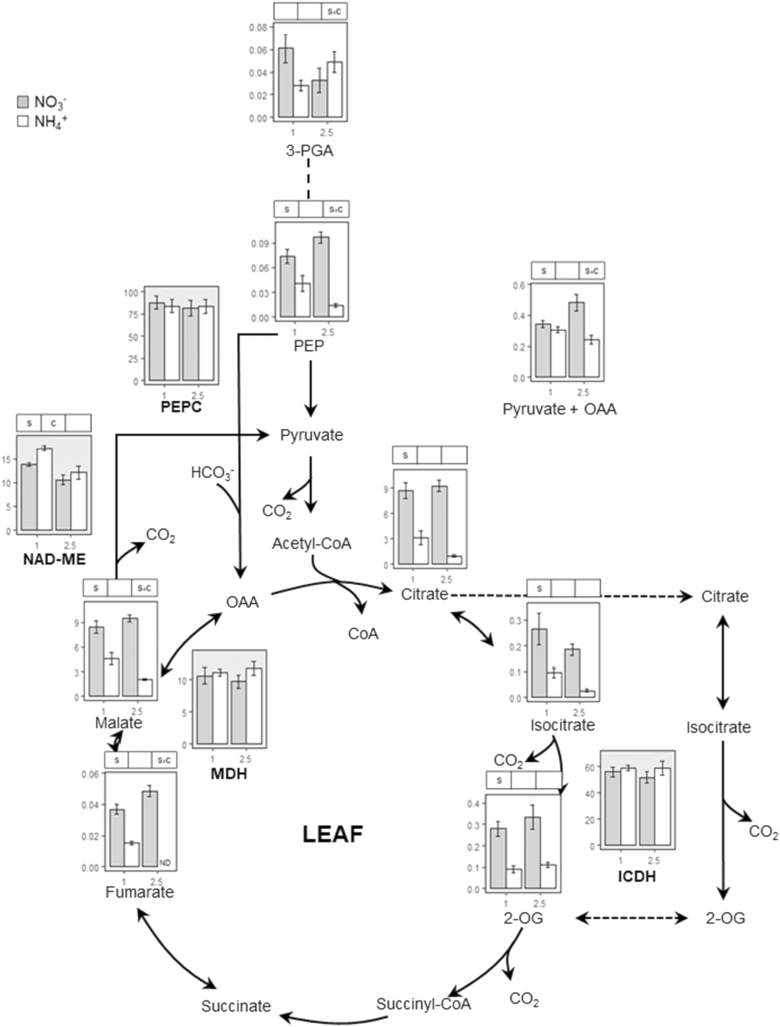
Organic acids and enzyme activities associate to TCA cycle in the leaf of *B. distachyon* grown for 24 days with nitrate or ammonium as N source at two different concentrations (1 and 2.5 mM). Organic acids content are given as nmol mg^−1^ FW. ICDH activity is given as μmol NADPH g^−1^ FW h^−1^, MDH as mmol NADH g^−1^ FW h^−1^ and ME, PEPC as μmol NADH g^−1^ FW h^−1^. Columns represent mean ± SE (*n* = 4). Significant differences according to two-way ANOVA are indicated by S for N source effects, C for N concentration effects and S × C for interactions (*P* < 0.05).

**Figure 6.**
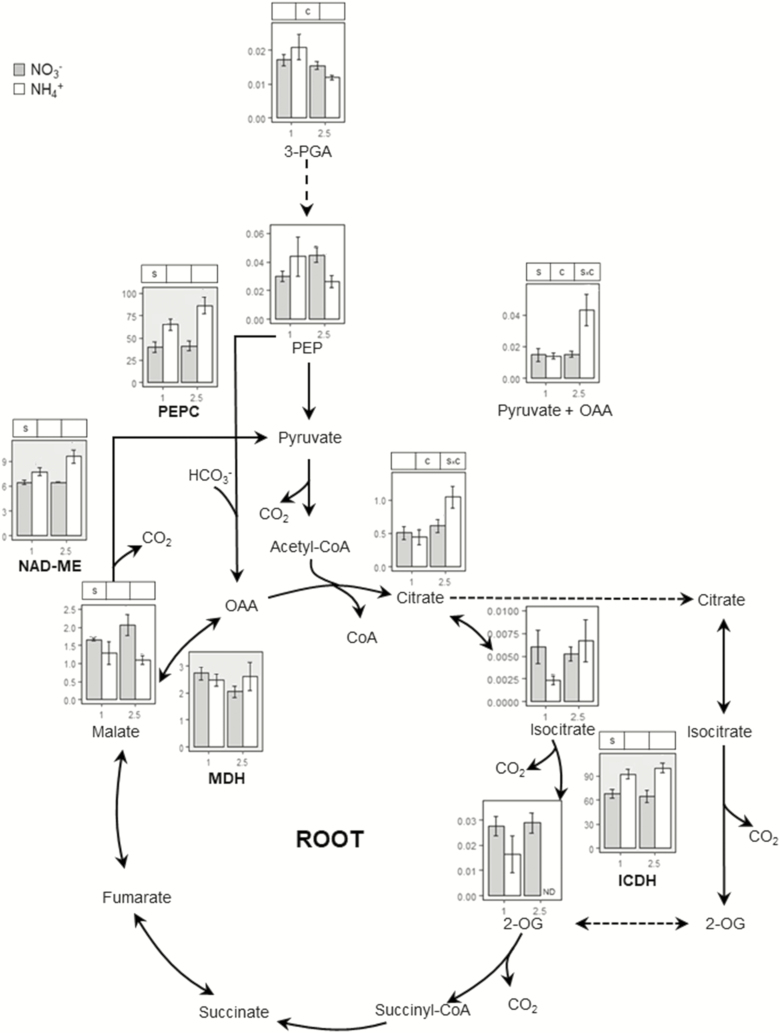
Organic acids and enzyme activities associate to TCA cycle in the root of *B. distachyon* grown for 24 days with nitrate or ammonium as N source at two different concentrations (1 and 2.5 mM). Organic acids content are given as nmol mg^−1^ FW. ICDH activity is given as μmol NADPH g^−1^ FW h^−1^, MDH as mmol NADH g^−1^ FW h^−1^ and ME, PEPC as μmol NADH g^−1^ FW h^−1^. Columns represent mean ± SE (*n* = 4). Significant differences according to two-way ANOVA are indicated by S for N source effects, C for N concentration effects and S × C for interactions (*P* < 0.05).

The TCA cycle and its associated pathways are essential to supply carbon skeletons for N assimilation, especially under ammonium nutrition. Thus, we determined the contents of relevant organic acids and the activity of a number of TCA-related enzymes (Figs 5 and 6; **see****Supporting Information—Fig. S2B**). In the leaf, no differences were observed in the activity of TCA-related enzymes, except for NAD-ME (Fig. 5; **see****Supporting Information—Fig. S2B**). Nevertheless, ammonium nutrition provoked a remarkable decrease in the content of each organic acid (Fig. 5). In roots, we observed higher activity of NADP-dependent isocitrate dehydrogenase (ICDH), NADP-dependent malic and phosphoenolpyruvate carboxylase (PEPC) enzymes in plants grown with ammonium supply (Fig. 6; **see****Supporting Information—Fig. S2B**). Access to nitrate or ammonium as N source brought about notable alterations in the content and distribution of organic acids in the root. Changes were mostly observed in 2.5 mM ammonium nutrition, where 3-PGA, PEP, 2-OG and malate contents were lower in plants grown with nitrate (Fig. 6). On the contrary, a significant source × concentration interaction was observed for pyruvate + oxaloacetate (Pyr + OAA) and citrate, whose levels were higher in plants fed with 2.5 mM ammonium compared to those fed with nitrate (Fig. 6). Finally, since ammonium has been shown by others to provoke an imbalance of essential cations, we assessed whether this was also the case for *B. distachyon*. In roots of plants grown with 2.5 mM of ammonium, Ca, Mg, and K contents were diminished compared to their nitrate counterparts **[see****Supporting Information—Table S4****]**. In leaves, K also decreased significantly in plants fed with ammonium **[see****Supporting Information—Table S4****]**.

## Discussion

Ammonium stress is considered universal in most if not all biological systems (Britto and Kronzucker 2002). However, great variability in ammonium-use efficiency has been reported (Britto and Kronzucker 2013; Sarasketa *et al.* 2014) and there are plant species and genotypes which display high tolerance of ammonium. Indeed, the Poaceae is considered to be relatively tolerant. For instance, ryegrass (*Lolium perenne*), wheat and notably rice are able to manage adequately the presence of high NH_4_^+^ concentrations in the external medium (Britto *et al.* 2001; Belastegui-Macadam *et al.* 2007; Setién *et al.* 2013). Additionally, intraspecific variability in ammonium toxicity has also been reported amongst different species, including cereals (Schortemeyer *et al.* 1997; Chen *et al.* 2013; Wang *et al.* 2016a).

*Brachypodium distachyon* has emerged as an excellent model species to study different aspects of development in C3 grasses and responses to a number of environmental constraints (Brutnell *et al.* 2015). However, studies of nitrogen metabolism remain scarce. Accordingly, we characterized the performance of Bd21, the reference accession for *B. distachyon*, supplied with nitrate or ammonium as N source with a special focus on interactions between carbon and nitrogen metabolisms.

*Brachypodium distachyon* Bd21 appeared to be moderately tolerant to ammonium. Indeed, in hydroponic culture it grew equally in 1 mM N regardless of N source. Importantly, 1 mM represented a N-sufficient condition, since raising NO_3_^−^ supply to 2.5 mM did not further increase plant biomass (Fig. 1). However, when NH_4_^+^ was raised to 2.5 mM plants displayed moderate symptoms of ammonium toxicity in terms of slower growth (Fig. 1).

A high NH_4_^+^ concentration in the medium is known to affect the homeostasis of essential cations in the cell. For instance, NH_4_^+^ affects K^+^ transport directly by competitive inhibition and indirectly via effects on membrane potential (Coskun *et al.* 2017). Moreover, NH_4_^+^ may also stimulate K^+^ efflux (Coskun *et al.* 2017). Among others, a decrease in K^+^ levels has been observed in grasses such as ryegrass (Belastegui-Macadam *et al.* 2007), rice (Balkos *et al.* 2010) or sorghum (Miranda *et al.* 2017). Indeed, when the available concentration of K^+^ in the nutrient solution is limiting, the provision of supplementary K^+^ improves ammonium tolerance (Balkos *et al.* 2010; Li *et al.* 2012). In contrast, this is not the case when the concentration of K^+^ is already sufficient (Balkos *et al.* 2010). In our work, despite the fact that we report a significant decrease in Ca, K and Mg content in the root of plants grown with 2.5 mM NH_4_^+^**[see****Supporting Information—Table S4****]**, the internal concentrations of these elements remained within the limits of an adequate nutrient supply (Marschner 2012). Therefore, under our growth conditions, ion imbalance does not seem to be the primary cause for the growth inhibition observed in *B. distachyon* fed with 2.5 mM NH_4_^+^. However, further experiments are needed to completely discard the potential role of cationic imbalance in the response of *B. distachyon* to ammonium toxicity.

The main metabolic symptom of ammonium toxicity, and the probable cause of subsequent disorders, is the disruption of cytosolic NH_4_^+^ homeostasis. Thus, one of the obvious cell strategies for keeping NH_4_^+^ cytosolic levels under control is to intensify its assimilation into organic molecules. It has already been reported that a prevalent response of many species, including grasses, is an accumulation of free amino acids primarily in the roots, e.g. in wheat and sorghum (Setién *et al.* 2013; Miranda *et al.* 2016). Alternatively, other species such as rapeseed (Coleto *et al.* 2017) tend to accumulate amino acids in shoot tissues. The amino acids in which NH_4_^+^ is preferentially stored also vary between species. As in other monocots such as wheat (Setién *et al.* 2013), NH_4_^+^ is scavenged in *B. distachyon* root systems in the form of amides, mainly Asn followed by Gln (Fig. 3).

Glutamine synthetase/glutamate synthase is the main NH_4_^+^ assimilation pathway and its importance during ammonium stress has been highlighted by the fact that mutants such as rice *gln1;1* or *Arabidopsis gln1;2* mutants show hypersensitivity to ammonium nutrition (Kusano *et al.* 2011; Guan *et al.* 2016). In *B. distachyon* Bd21, GS is encoded by four genes **[see****Supporting Information—Table S1**, **Fig. S4****]**. Various studies have revealed that GS is regulated at multiple levels: transcriptional, translational and post-translational. Moreover, GS members are differentially regulated (i) in space, according to cell type and function of the organ; (ii) in time, according to circadian rhythm and phenology; and (iii) in response to growth conditions (Swarbreck *et al.* 2011). Accordingly and in contrast to the decrease of GS activity observed in roots of plants fed with ammonium (Fig. 3), the expression of *BdGLN1;1* and *BdGLN1;2* did not vary and only *BdGLN1;3* expression was enhanced by NH_4_^+^ (Fig. 4A) thereby underlining the probable post-translational regulation of GS. A further and interesting finding is that in roots of barley, *HvGS1.3*, homologue of *BdGLN1;3* was also induced by NH_4_^+^ nutrition (Goodall *et al.* 2013), indicating that the function and regulation of these isogenes can be similar for both monocot species. Thus, it will be of interest to explore whether the role of *BdGLN1;3* is significant under ammonium stress. The decrease in root GS activity observed in plants fed with ammonium (Fig. 3) could be related to a feedback regulation by the accumulation of amino acids (Watanabe *et al.* 1997; Oliveira and Coruzzi 1999) as represented by the high Asn/Asp and Gln/Glu ratios (Fig. 3; **see****Supporting Information—Fig. S3**, **Table S3**).

Asparagine synthetase (AS) catalyses the ATP-dependent synthesis of Asn and Glu from Gln and Asp. Asn has a high ratio N:C and plays a key role in nitrogen transport and storage (Lea *et al.* 2007). *BdASN1* and *BdASN3* were induced by ammonium treatment (Fig. 4C) and thus, probably control Asn synthesis in *B. distachyon* fed with ammonium. Indeed, in plants fed with 2.5 mM NH_4_^+^, Asn accounted for *ca*. 51 and 70 % of total leaf and root amino acid content, respectively **[see****Supporting Information—Tables S2****and****S3****]**. The higher expression of *BdASN1* and its specific up-regulation by ammonium nutrition in roots suggest a prominent role of this gene when ammonium is the only source of N (Fig. 4C). In support of this, *OsAS1*, a homologue of *BdASN1***[see****Supporting Information—Fig. S6****]**, was previously shown responsible for Asn accumulation in rice grown under ammonium nutrition (Ohashi *et al.* 2015).

To sustain amino acid synthesis in the root an adequate supply of carbon is essential, necessitating adjustments in carbon metabolism elsewhere. Specifically, TCA cycle adjustment has been shown to be crucial, as well as its associated anaplerotic routes (Setién *et al.* 2014; Sarasketa *et al.* 2016). The significant effect of ammonium nutrition on PEPC activity (Fig. 6) was presumably essential to guarantee the flux of carbon towards OAA that can yield Asp or follow the Krebs cycle. Moreover, ME activity would also collaborate, through pyruvate entrance in the cycle, in the provision of citrate + isocitrate to generate 2-OG in co-ordination with ICDH (Fig. 6). Indeed, low malate and PEP contents indicate a higher consumption under ammonium nutrition; therefore, confirming the prioritization of the pathway generating TCA intermediates, mainly OAA and 2-OG. The role of PEPC, ME and ICDH in root response to ammonium nutrition has been highlighted in different species including pea and sorghum (Ariz *et al.* 2013; Arias-Baldrich *et al.* 2017). Moreover, the relevance of PEPC agrees with a report where *Arabidopsis ppc1/ppc2* double mutant was impaired in ammonium assimilation (Shi *et al.* 2015). Complementary evidence that the plant is prioritizing carbon provision to the roots is also evidenced by a general decrease of leaf organic acids (Fig. 5) and additionally of Glu and Asp (Fig. 2). As a consequence, we observed a clear N source and concentration effect in total root C content that increased in plants grown with ammonium and provoked a decrease in C:N ratio compared to plants fed only with nitrate **[see****Supporting Information—Fig. S7****]**.

The enhancement of GDH activity in relation with the up-regulation of *GDH2* gene expression is one of the best metabolic markers of ammonium nutrition. Indeed, this GDH response has been reported in different monocots including wheat (Setién *et al.* 2013; Wang *et al.* 2016b), rice (Xuan *et al.* 2013) and *B. distachyon* in the present study (Figs 2–4). It has been hypothesized that GDH induction could be related to direct NH_4_^+^ assimilation because of GDH aminating activity and there are published hints for such a role (Skopelitis *et al.* 2006). However, other evidence also supports the idea that the role of GDH is to deaminate glutamate, thus collaborating in 2-OG provision (Labboun *et al.* 2009). We also report higher levels of NADP-GDH activity in *B. distachyon* fed with ammonium **[see****Supporting Information—Fig. S1A****]**. This enzyme is still poorly characterized in plants compared to GDH. Whether NAD(H)- and NADP(H)-dependent GDH enzymes possess differential functions in plant metabolism in general and in relation with ammonium nutrition in particular remains to be elucidated.

In conclusion, we report that *B. distachyon* Bd21 is a species with moderate tolerance to ammonium nutrition. We observed a strong metabolic adaptation of *B. distachyon* carbon and nitrogen metabolism when facing ammonium-only N nutrition. The root system is shown as a physiological barrier acting as a reservoir for free NH_4_^+^ and increasing NH_4_^+^ assimilation to amides. This was possible since TCA enzyme activities together with anaplerotic routes were adjusted to increase 2-OG and OAA provision thereby sustaining Gln and Asn synthesis in the root. This metabolic adjustment appears to be a strategy mitigating ammonium stress while imposing an energetic cost for the cell that limits plant growth under 2.5 mM NH_4_^+^ supply. Overall, these responses of *B. distachyon* to ammonium nutrition are in line with previous studies with cereals crops. Our work underlines the potential of *B. distachyon* as a useful tool for analysing the molecular basis of ammonium tolerance in monocots. Such work is of paramount importance in view of the desirability of increasing the use of ammonium-based fertilizers to lessen environmental pollution associated with nitrate-based nutrition.

## Data

The raw data that support the findings of this study are available from the corresponding author, upon reasonable request.

## Sources of Funding

This research was funded/supported by the Basque Government (IT932-16) and the Spanish Ministry of Economy and Competitiveness (BIO2017-84035-R co-funded by FEDER).

## Contributions by the Authors

D.M. and M.B.G. conceived the study and supervised the project; M.P. and D.M. performed experiments. M.P., M.B.G and D.M. analysed data. M.P. and D.M. wrote the paper and, all authors edited and approved the final manuscript.

## Conflict of Interest

None declared.

## Supplementary Material

plz029_suppl_Supporting_Information

## Supporting Information

The following additional information is available in the online version of this article—

**Figure S1.** Nitrate contents in leaves and roots of *Brachypodium distachyon* grown with nitrate or ammonium as N source.

**Figure S2.** NADP(H)-GDH (A) and NADP-dependent ME (B) enzyme activities in leaves and roots of *Brachypodium distachyon* grown with nitrate or ammonium as N source.

**Figure S3.** Gln/Glu (A) and Asn/Asp (B) ratios in leaves and roots of *Brachypodium distachyon* grown with nitrate or ammonium as N source.

**Figure S4.** Phylogenetic tree of glutamine synthetase (*GS*) genes.

**Figure S5.** Phylogenetic tree of glutamate dehydrogenase (*GDH*) genes.

**Figure S6.** Phylogenetic tree of asparagine synthetase (*ASN*) genes.

**Figure S7.** C:N ratio (A) and carbon content (B) of leaves and roots of *Brachypodium distachyon* grown with nitrate or ammonium as N source.

**Table S1.** *Brachypodium distachyon* Bd21 genes encoding for GLN, AS and GDH enzymes.

**Table S2.** Individual amino acid contents in leaves of *Brachypodium distachyon* grown with nitrate or ammonium as N source.

**Table S3.** Individual amino acid contents in roots of *Brachypodium distachyon* grown with nitrate or ammonium as N source.

**Table S4.** K, Ca, Mg and Na contents of leaves and roots of *Brachypodium distachyon* grown with nitrate or ammonium as N source.
