# Supplementary material for: Providing carbon skeletons to sustain amide synthesis in roots underlines the suitability of Brachypodium distachyon for the study of ammonium stress in cereals
Source: AoB Plants. 2019 May 10;11(3):plz029. doi: 10.1093/aobpla/plz029 (PMC6534281; doi:10.1093/aobpla/plz029)
Supplement: plz029_suppl_Supporting_Information [file plz029_suppl_supporting_information.pdf]

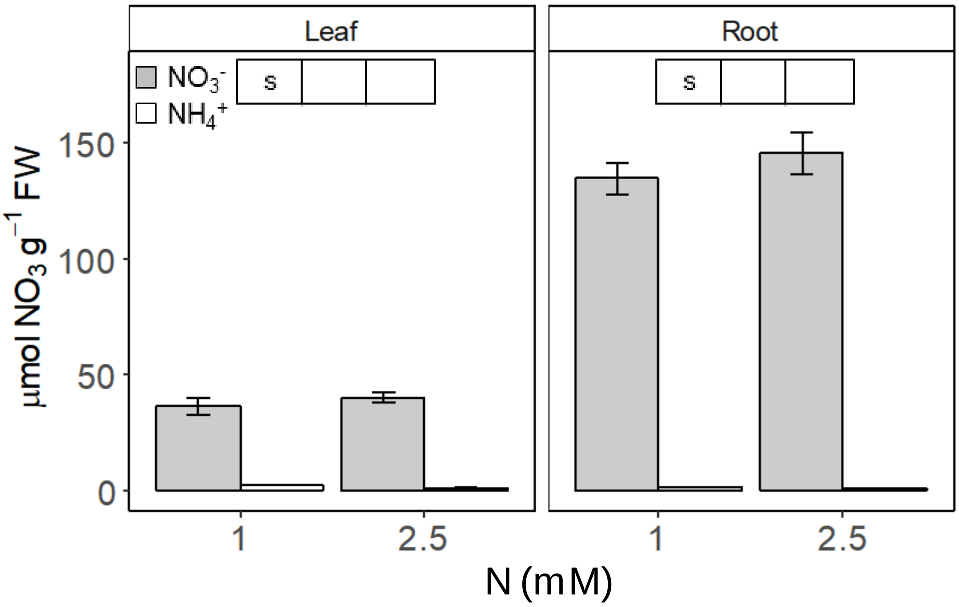

Figure S1. Nitrate content in leaf and root of *B. distachyon* Bd21 grown for 24 days with 1 or 2.5 mM of nitrate or ammonium as N source. Values represent mean ± SE (n = 4). Significant differences according to two-way ANOVA are indicated by S for N source effects, C for N concentration effects and SxC for interactions ($P < 0.05$)**.**

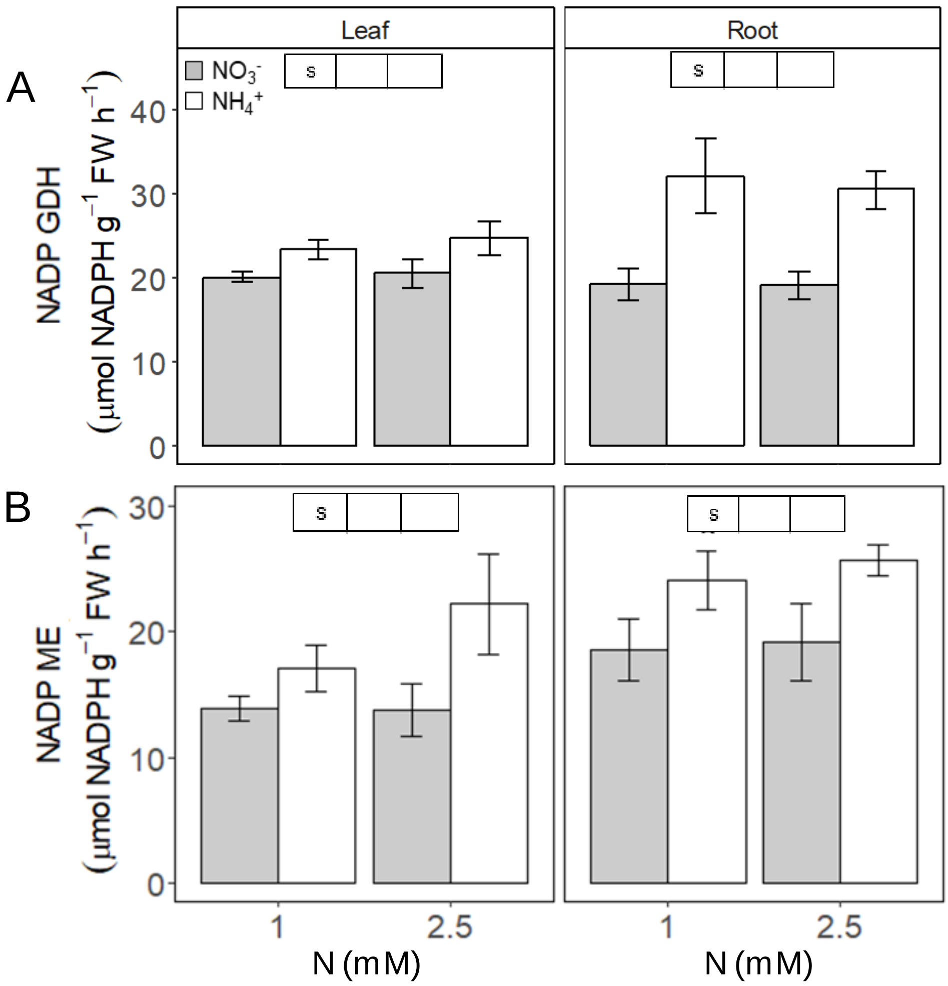

Figure S2. NADP-GDH and NADP-dependent ME enzyme activities (B) in leaf and root of *B. distachyon* Bd21 grown for 24 days with 1 or 2.5 mM of nitrate or ammonium as N source. (A) NADP-GDH enzyme activity. **(B)** NADP-ME enzyme activity. Values represent mean ± SE (n = 4). Significant differences according to two-way ANOVA are indicated by S for N source effects, C for N concentration effects and SxC for interactions (P < 0.05).

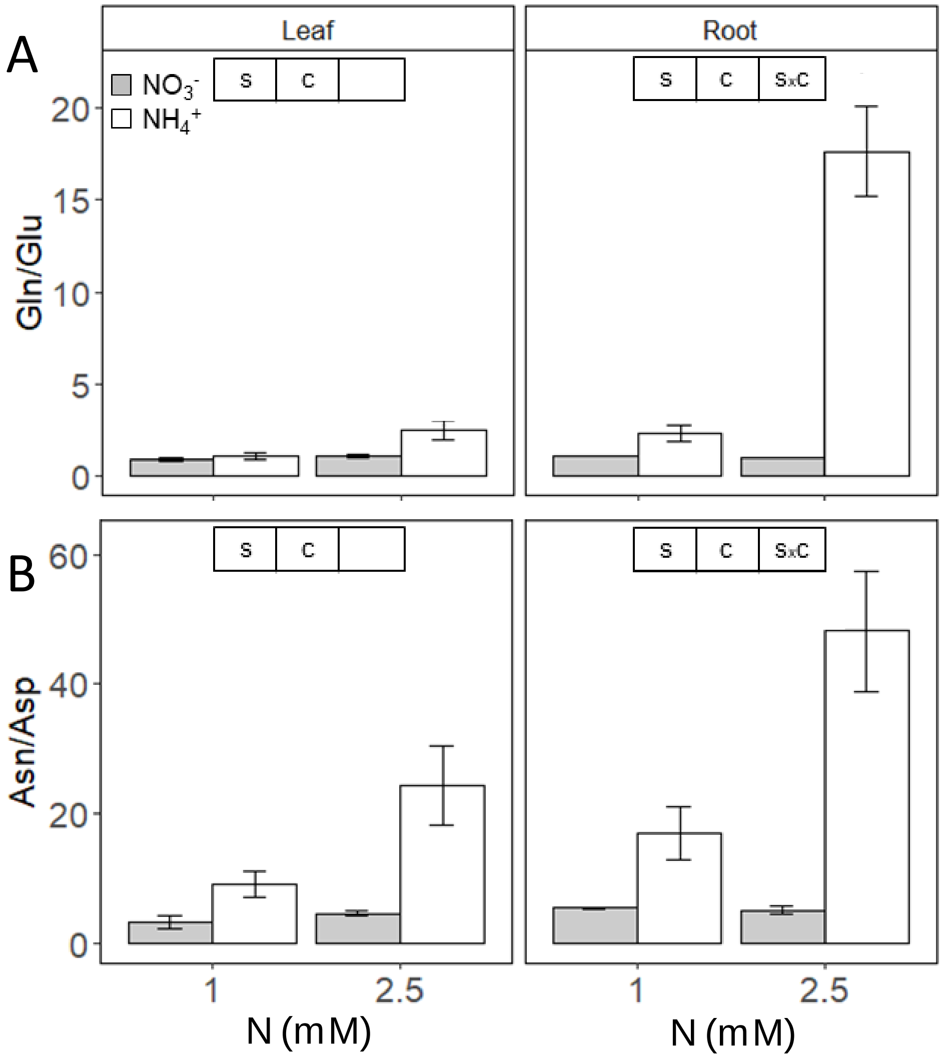

Figure S3. Gln/Glu and Asn/Asp ratio of leaf and root of *B. distachyon* grown for 24 days with nitrate or ammonium as N source. (A) Gln/Glu ratio. **(B)** Asn/Asp ratio. Values represent mean ± SE (n = 3). Significant differences according to two-way ANOVA are indicated by S for N source effects, C for N concentration effects and SxC for interactions ($P < 0.05$)**.**

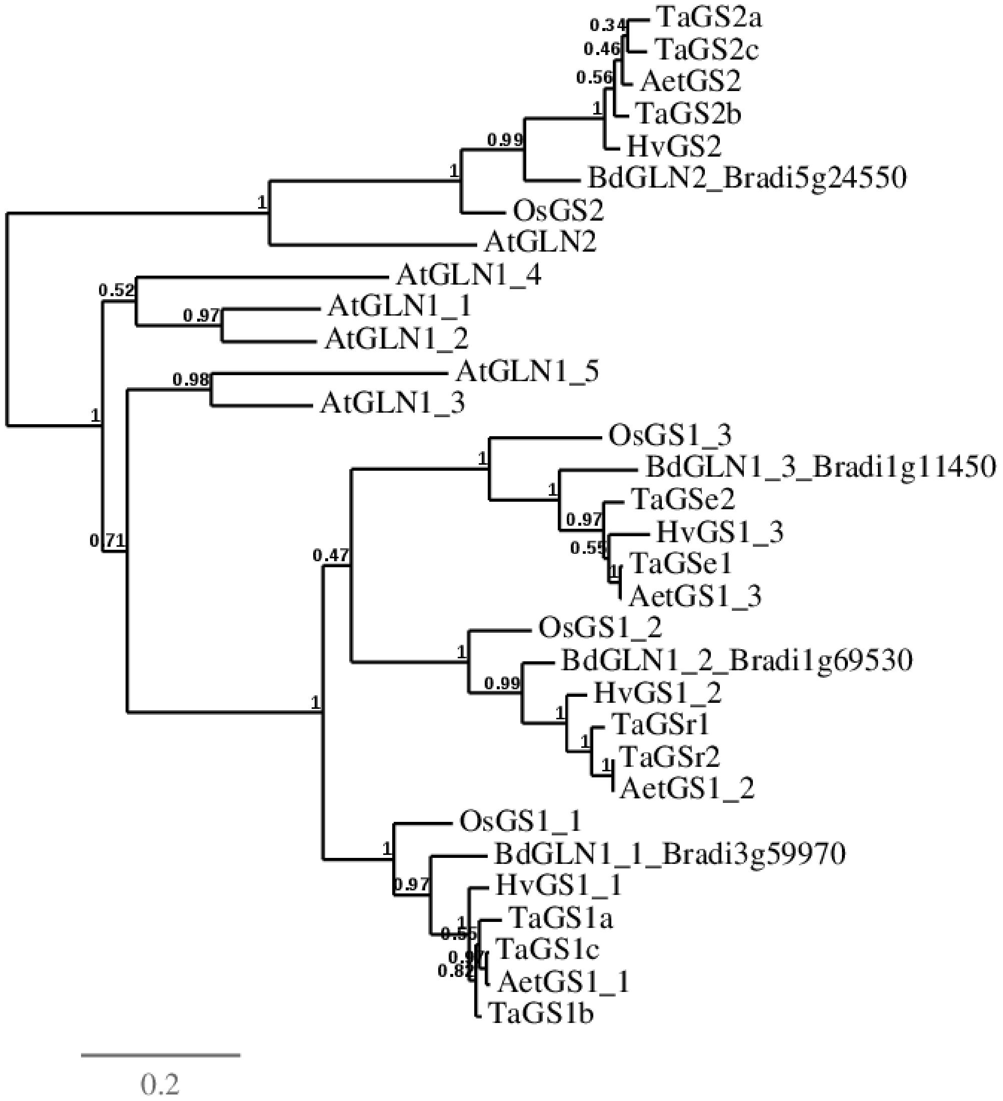

Figure S4. Phylogenetic tree of glutamine synthetase genes.

Six plant species were used *Oryza sativa* (Os), *Arabidopsis thaliana* (At), *Aegilops tauchii* (Aet), *Hordeum vulgare* (Hv), *Triticum aestivum* (Ta), and *Brachypodium distachyon* (Bd). The genes used for the analysis are *OsGS1;1* (AB037595), *OsGS1;2* (AB180688), *OsGS1;3* (AB180689), *OsGS2* (X14246), *AtGLN1;1* (At5g37600), *AtGLN1;2* (At1g66200), *AtGLN1;3* (At3g17820), *AtGLN1;4* (At5g16570), *AtGLN1;5* (At1g48470), *AtGLN2* (At5g35630), AetGS1;1 (XM_020314490) AetGS1;2 (XM_020336960), AetGS1;3 (XM_020323172), AetGS2 (XM_020309033), *HvGS1;1* (JX878489), *HvGS1;2* (JX878490), *HvGS1;3* (JX878491), *HvGS2* (AK360336), *TaGS1a* (DQ124209), *TaGS1b* (DQ124210), *TaGS1c* (DQ124211), *TaGSr1* (AY491968), *TaGSr2* (AY491969), *TaGSe1* (AY491970), *TaGSe2* (AY491971), *TaGS2a* (DQ124212), *TaGS2b* (DQ124213), *TaGS2c* (DQ124214). Bd genes are shown in the tree with their respective codes. Numbers indicate branch support values.

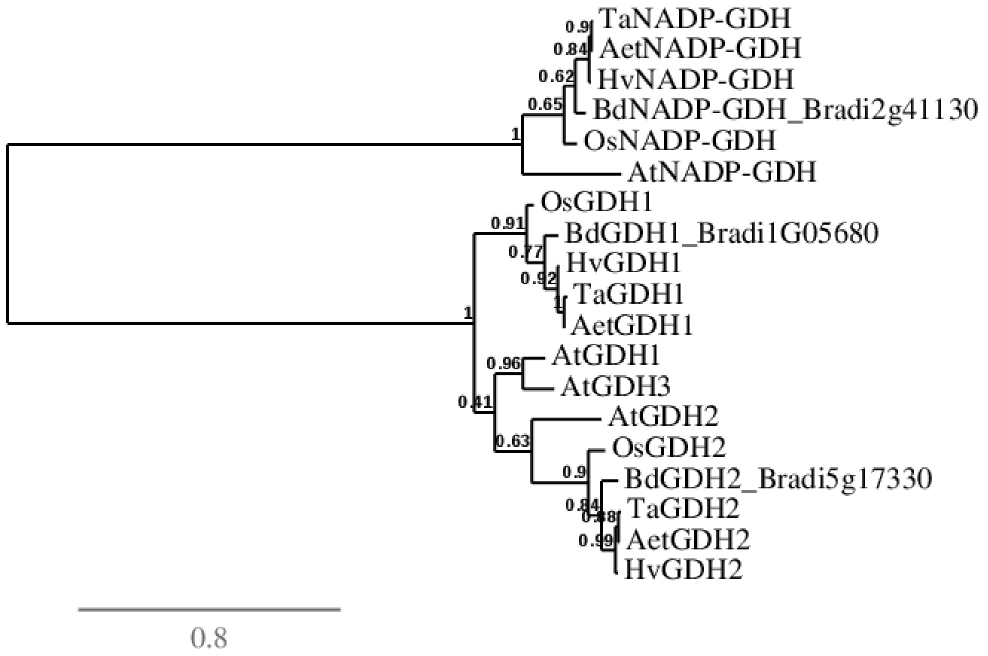

Figure S5. Phylogenetic tree of glutamate dehydrogenase genes.

Six plant species were used *Oryza sativa* (Os), *Arabidopsis thaliana* (At), *Aegilops tauchii* (Aet), *Hordeum vulgare* (Hv), *Triticum aestivum* (Ta), and *Brachypodium distachyon* (Bd). The genes used for the analysis are *OsGDH1* (AB024962), *OsGDH2* (AB189166), *OsNADP-GDH* (XM_015764008), *AtGDH1* (At1g51720), *AtGDH2* (At5g07440), *AtGDH3* (At3g03910), *AtNADP-GDH* (At1g51720), *AetGDH1* (XM_020327456), *AetGDH2* (XM_020324446), *AetNADP-GDH* (XM_020298560), *HvGDH1* (AK369720), *HvGDH2* (AK366146), *HvNADP-GDH* (AK369992). For wheat a gene representative of each GDH type was selected *TaGDH1 (*AK449125*)*, *TaGDH2* (AK331666) and *TaNADP-GDH* (AK455316). Bd genes are shown in the tree with their respective codes. Numbers indicate branch support values.

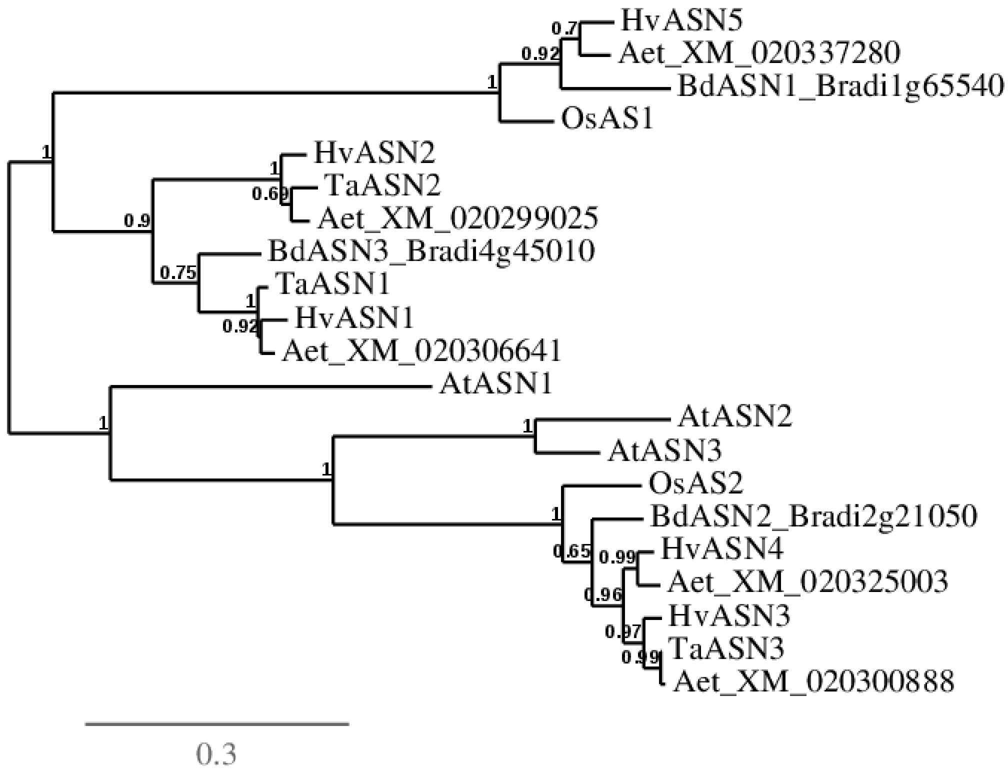

Figure S6. Phylogenetic tree of asparagine synthetase genes.

Six plant species were used *Oryza sativa* (Os), *Arabidopsis thaliana* (At), *Aegilops tauchii* (Aet), *Hordeum vulgare* (Hv), *Triticum aestivum* (Ta), and *Brachypodium distachyon* (Bd). The genes used for the analysis are *OsAS1* (XM_015776603), *OsAS2* (XM_015787697), *AtASN1* (At3g47340), *AtASN2* (At5g65010), *AtASN3* (At5g10240), *HvASN1* (AK359770), *HvASN2* (AK357350), *HvASN3* (AK353762), *HvASN4* (AK363899), *HvASN5* (AK361923), *TaASN2* (KY937996), *TaASN1* (KY937995), *TaASN3* (KY937997), the five *AetASN* genes found in the databases are shown with their gene codes in the tree. Bd genes are shown in the tree with their respective codes. Numbers indicate branch support values.

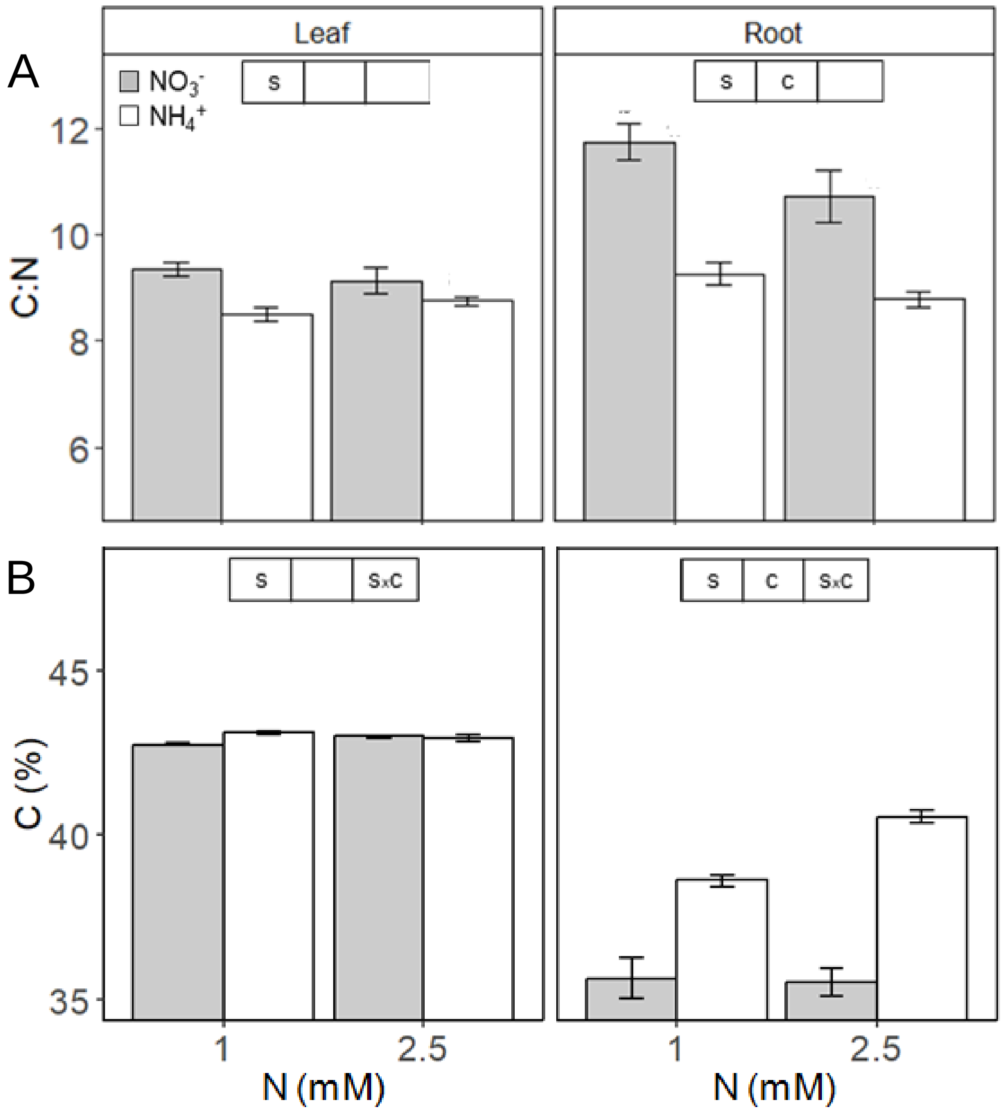

Figure S7. C:N ratio and carbon content of leaf and root of *B. distachyon* grown for 24 days with nitrate or ammonium as N source. (A) C:N ratio. **(B)** Carbon content. Values represent mean ± SE (n = 4). Significant differences according to two-way ANOVA are indicated by S for N source effects, C for N concentration effects and SxC for interactions ($P < 0.05$)**.**

Table S1. ***Brachypodium distachyon*** **Bd21 genes encoding for GLN, AS and GDH enzymes.**

Name	Locus name	Gene symbol	Forward	Reverse	E
BdGLN1;1	Bradi3g59970	LOC100845598	aagctgcccaagtggaactacg	tccttgaagatagcctgtgggtag	1.76
BdGLN1;2	Bradi1g69530	LOC100824429	tcgtcgagtacttgtgggttgg	ccattcacggtccttgctttgc	1.80
BdGLN1;3	Bradi1g11450	LOC100837122	caagccatcttcagggatccattc	tgcatagcagtcacacataaccag	1.92
BdGLN2	Bradi5g24550	LOC100842712	tcgcttcacaagtgccatggtatg	agaggccagttcacatctctctgg	1.76
BdASN1	Bradi1g65540	LOC100830770	tgccgaagcatatcctctac	tttcatcatctcatcggtgac	1.93
BdASN2	Bradi2g21050	LOC100830419	tctcttgtctggtggacttg	tcaggagaacccttcaaacc	2.02
BdASN3	Bradi4g45010	LOC100839462	cagtgttcaggatggcattg	gcgacttgatcttgcgtgac	2.05
BdGDH1	Bradi1g05680	LOC100831066	agtttcatggttactcgcctgctg	tcccagagatcctccaaggtcaac	1.89
BdGDH2	Bradi5g17330	LOC100828452	acatgggaactaatgcacagacc	agtggcagcatccctacctaatg	1.91
BdNADP-GDH	Bradi2g41130	LOC100833076	cgatgccgatctacgtcaaagc	tgaaccacctcctggatagactg	1.95
BdSamDC	Bradi5g14640	LOC100821874	tgctaatctgctccaatggc	gacgcagctgaccacctaga	2.04
BdACT3	Bradi4g41850	LOC100834364	cctgaagtccttttccagcc	agggcagtgatctccttgct	2.05

The primers used for qPCR expression together with their efficiencies (E) are shown. *BdSamDC* and *BdACT3* are the genes that served as reference for relative gene expression quantification.

Table S2. Individual amino acid content (µmol . g FW^{-1}) of leaf of *B. distachyon* grown for 24 days with nitrate or ammonium as N source.

	Leaf				
	1 mM		2.5 mM		Variance analysis
	NO_3^-	NH_4^+	NO_3^-	NH_4^+	
**Ala**	3.16 ± 0.21	3.84 ± 0.43	3.6 ± 0.15	3.32 ± 0.17	
**Arg**	0.15 ± 0.03	0.51 ± 0.17	0.24 ± 0.03	0.91 ± 0.26	S
**Asn**	8.42 ± 3.3	20.3 ± 4.88	13.17 ± 0.86	28.74 ± 6.84	S
**Asp**	2.43 ± 0.45	2.17 ± 0.32	2.85 ± 0.13	1.19 ± 0.08	S, SxC
**GABA**	0.4 ± 0.05	0.61 ± 0.07	0.44 ± 0.03	0.49 ± 0.08	
**Gln**	6.2 ± 1.41	6.91 ± 1.39	8.82 ± 0.63	11.44 ± 2.17	C
**Glu**	6.94 ± 1.01	6.44 ± 0.67	8.11 ± 0.13	4.72 ± 0.26	S, SxC
**His**	0.11 ± 0.01	0.13 ± 0.02	0.12 ± 0.01	0.12 ± 0.02	
**Ile**	0.06 ± 0.01	0.08 ± 0.01	0.07 ± 0.00	0.10 ± 0.02	S
**Leu**	0.09 ± 0.01	0.11 ± 0.02	0.10 ± 0.00	0.16 ± 0.03	
**Lys**	0.09 ± 0.03	0.23 ± 0.07	0.13 ± 0.01	0.46 ± 0.15	S
**Met**	0.06 ± 0.02	0.07 ± 0.01	0.09 ± 0.01	0.06 ± 0.01	
**Phe**	0.08 ± 0.01	0.09 ± 0.02	0.10 ± 0.00	0.10 ± 0.02	
**Ser-Gly**	2.38 ± 0.09	2.67 ± 0.14	2.58 ± 0.06	2.90 ± 0.30	
**Thr**	1.52 ± 0.14	1.52 ± 0.25	1.71 ± 0.05	1.26 ± 0.10	
**Trp**	0.01 ± 0.00	0.01 ± 0.00	0.01 ± 0.00	0.03 ± 0.00	S, C, SxC
**Tyr**	0.12 ± 0.01	0.12 ± 0.03	0.14 ± 0.01	0.16 ± 0.02	
**Val**	0.23 ± 0.02	0.25 ± 0.02	0.24 ± 0.01	0.35 ± 0.06	

Values represent mean ± SE (n = 3). The column "Variance analysis" shows significant differences according to two-way ANOVA. S indicates N source effects, C N concentration effects and SxC interactions $P < 0.05$)**.**

Table S3. Individual amino acid content (µmol . g FW^{-1}) of root of *B. distachyon* grown for 24 days with nitrate or ammonium as N source.

	Root				
	1 mM		2.5 mM		Variance
	NO_3^-	NH_4^+	analysis	NH_4^+	analysis
**Ala**	1 ± 0.07	1.43 ± 0.16	0.93 ± 0.02	1.64 ± 0.36	S
**Arg**	0.12 ± 0.01	0.12 ± 0.02	0.1 ± 0.01	0.32 ± 0.07	S, C, SxC
**Asn**	3.85 ± 0.19	13.89 ± 2.9	2.93 ± 0.76	48.47 ± 10.28	S, C, SxC
**Asp**	0.7 ± 0.01	0.83 ± 0.08	0.54 ± 0.08	1.01 ± 0.14	S
**GABA**	0.52 ± 0.06	0.77 ± 0.13	0.42 ± 0.05	0.71 ± 0.04	S
**Gln**	1.49 ± 0.03	3.23 ± 0.51	1.09 ± 0.14	20.37 ± 2.97	S, C, SxC
**Glu**	1.44 ± 0.04	1.43 ± 0.05	1.16 ± 0.16	1.17 ± 0.15	C
**His**	0.06 ± 0.01	0.06 ± 0.01	0.06 ± 0.01	0.2 ± 0.06	SxC
**Ile**	0.12 ± 0.01	0.12 ± 0.00	0.10 ± 0.00	0.22 ± 0.03	S, C, SxC
**Leu**	0.16 ± 0.01	0.16 ± 0.01	0.14 ± 0.00	0.3 ± 0.03	S, C, SxC
**Lys**	0.07 ± 0.00	0.06 ± 0.01	0.06 ± 0.00	0.23 ± 0.08	
**Met**	0.09 ± 0.00	0.05 ± 0.01	0.07 ± 0.01	0.04 ± 0.01	S
**Phe**	0.04 ± 0.00	0.05 ± 0.00	0.03 ± 0.00	0.15 ± 0.02	S, C, SxC
**Ser-Gly**	0.74 ± 0.00	0.8 ± 0.06	0.67 ± 0.04	1.25 ± 0.02	S, C, SxC
**Thr**	0.69 ± 0.01	0.61 ± 0.04	0.59 ± 0.03	0.92 ± 0.03	S, C, SxC
**Trp**	0.04 ± 0	0.04 ± 0.01	0.04 ± 0.00	0.1 ± 0.02	S, C, SxC
**Tyr**	0.08 ± 0.01	0.05 ± 0.01a	0.07 ± 0.00	0.17 ± 0.02	S, C, SxC
**Val**	0.56 ± 0.03	0.59 ± 0.04	0.41 ± 0.03	0.94 ± 0.1	S, SxC

Values represent mean ± SE (n = 3). The column "Variance analysis" shows significant differences according to two-way ANOVA. S indicates N source effects, C N concentration effects and SxC interactions $P < 0.05$).

Table S4. K, Ca, Mg and Na content (mg. g DW^{-1}) of roots and leaves of *B. distachyon* grown for 24 days with 2.5 mM nitrate or ammonium as N source.

	Leaf		Root	
	NO_3^-	NH_4^+	NO_3^-	NH_4^+
**Ca**	6.80 ± 0.50	6.62±1.15	**21.31 ± 4.66**	**5.62 ± 2.03**
**K**	**42.23 ± 0.46**	**30.29±3.68**	**58.27 ± 4.53**	**24.24 ± 2.39**
**Mg**	2.46 ± 0.11	2.81±0.26	**4.65 ± 0.14**	**2.11 ± 0.23**
**Na**	0.22 ± 0.02	0.21 ± 0.02	0.44 ± 0.04	0.45 ± 0.06

Values represent mean ± SE (n = 4). Significant nitrogen source effect within each dose (t-test, $p < 0.05$) is highlighted in bold.
